# Sources of Oral Health Activities Among Croatian University Students—A Pilot Study

**DOI:** 10.3390/dj14030146

**Published:** 2026-03-05

**Authors:** Diana Aranza, Tina Poklepović Peričić, Boris Milavić

**Affiliations:** 1 Faculty of Health Sciences, University of Split, 21000 Split, Croatia; 2 Division of Maxillofacial Surgery, Subdivision of Dental Medicine, University Hospital of Split, 21000 Split, Croatia; tina.poklepovic.pericic@mefst.hr; 3 Department of Prosthodontics, Study of Dental Medicine, School of Medicine, University of Split, 21000 Split, Croatia; 4 Faculty of Kinesiology, University of Split, 21000 Split, Croatia

**Keywords:** dental care, dental health services, health knowledge, health youth, oral health education, oral health literacy, oral health related, oral health surveys, oral hygiene

## Abstract

**Background:** This cross-sectional designed study aimed to identify the sources of oral health activities (OHA) by introducing a new OHA sources questionnaire (OHAQ-S). **Methods:** The OHAQ-S was developed from a sample of 658 university students and included measurements from nine sources: scales for *parents*, *Dental medical doctors (DMDs)*, and *primary school* sources, as well as single-item measures for other sources. Using QHAQ-S measures, gender differences, determinants of OH activities, and differences between *OH types* were analysed. **Results:** Gender differences were observed in five OH sources (*university, high school*, *self-learning*, *friends,* and *kindergarten*). In both female and male subsamples, *primary sources* such as *parents* and *DMD* predicted overall OH activities, though with different secondary sources. In the female subsample, some differences in OHAQ-S sources appeared between the four OH types. The *excellent OH type* most notably differed from others by having higher reported incidence of *self-learning—dental floss usage* and *DMD sources*—and marginally higher reported use of *university* and *parental sources*. In the male subsample, multiple differences in OHAQ-S sources were found among the four OH types. The *excellent OH type* most distinguished itself by reporting higher levels of *DMD*, *self-learning—dental floss usage*, *university—acquired OH knowledge*, *parental*, *and media and internet—health journal sources*. **Conclusions:** Female students have differently expressed and more-pronounced OHA sources relative to male students and some sources encountered earlier (*kindergarten* and *high school* sources), and “independent” learning sources (*self-learning* versus *friends sources*). In both subsamples, predictive relationships of OHAQ-S measures with *overall OH activities* were verified. The findings on the elements of the discriminative and predictive validity of the pilot version of the questionnaire show that the OHAQ-S questionnaire represents a quality basis for constructing a questionnaire on sources of OH activities.

## 1. Introduction

### 1.1. Information Sources and Oral Health Behaviours Among Young People

Oral health is a vital aspect of a person’s overall health and well-being, one closely connected to physical, emotional, and social functioning. The World Health Organisation (WHO) defines oral health as a state in which an individual is free from pain in the mouth; infections; or diseases that impair their ability to chew, speak, smile, or participate in social interactions [1]. During adolescence, a critical developmental period for forming habits, attitudes, and behaviours, oral hygiene has enduring effects on health [2]. Oral health is a key component of overall health and significantly impacts quality of life, especially in childhood and adolescence, when health habits are established [2]. Young people are particularly vulnerable to external influences such as socioeconomic conditions, peer pressure, education systems, and digital media, which shape their oral hygiene practices [3,4].

Despite the widespread availability of preventive measures globally, the prevalence of oral diseases among young people remains high, even though caries and periodontal disease are preventable through proper hygiene and regular dental visits [5]. In certain regions, like middle- and low-income countries, the incidence of caries in children surpasses 80% [6]. Variations are also notable within developed nations, where factors influencing rising oral disease rates include socioeconomic status, parental education, and access to preventive services [7,8]. Caries and gingivitis are among the most common chronic diseases affecting children and adolescents worldwide, with over 60% of adolescents having experienced caries [1,7]. Significant disparities in oral hygiene habits are observed across Europe and Asia, determined by levels of socioeconomic status and access to education [9,10,11,12].

The oral health behaviour of young people is strongly influenced by parental supervision, health literacy, access to dental care, and school-based education [8]. Research indicates that adolescents who regularly visit the dentist and participate in structured educational programmes are more likely to maintain good oral hygiene and use supplementary tools such as dental floss and mouthwash [13].

Oral health education that includes interactive methods and early intervention greatly encourages the development of healthy habits. Randomised controlled trials conducted in schools and kindergartens show that short, consistent education sessions with demonstrations reduce plaque and raise the frequency of brushing and flossing [14]. Interventions based on social–cognitive theory and involving parents further improve programme effectiveness [3,15].

Additionally, the international HBSC (Health Behaviour in School-aged Children) study found that adolescents from higher socioeconomic backgrounds tend to brush their teeth twice daily and consume fewer sugary drinks [9]. Similar findings are supported by research in Asia, which indicates that dietary habits and sugar intake are strong predictors of oral health [11,12]. In this context, schools and educational institutions function as key intervention points. The evidence suggests that structured, interactive school programmes, including demonstrations of proper oral hygiene, nutrition education, and regular check-ups, can significantly improve students’ knowledge, attitudes, and behaviours [13,14,16,17]. The most effective outcomes are achieved when parents and health professionals, particularly dentists, are involved, as their participation enhances children’s motivation and understanding [15].

Alongside environmental factors, social–cognitive elements also play a vital role in shaping oral hygiene practices. According to Bandura’s social learning theory, behaviour is learned through observing models in the immediate environment—such as peers, parents, and the media—and through perceived benefits and reinforcements [3]. During adolescence, peers become particularly influential, and research shows that social circles significantly influence perceptions of aesthetics, the importance of oral hygiene, and the necessity for regular check-ups [4]. In public health, an increasing body of research highlights the role of information sources in shaping adolescents’ health behaviours. Oral hygiene, although a simple practice, requires motivation, understanding, and consistency, and the type and quality of information young people access play a key role in their behaviour. Young people usually receive information about oral health from their parents, and through these three primary sources: (i) dental professionals, (ii) educational programmes in kindergartens and schools, and (iii) digital media, including social media [18,19]. The impact of parents on children’s oral health has been confirmed in many studies [20].

Children of carers with low oral health literacy tend to have harmful oral health habits, such as inadequate tooth brushing and nighttime bottle use [21,22]. It has been determined that dental caries is more common among children whose parents have low oral health literacy [23,24]. Digital tools, such as educational applications and animated content, have great potential to motivate young users, track their progress, and strongly influence their oral hygiene behaviour. At the same time, developing media literacy is essential to enable children and adolescents to recognise false or harmful health messages online [5]. The ability to critically evaluate health claims and make responsible health decisions is crucial for individuals [25,26] which means recognising and assessing which health claims are reliable and beneficial to oneself.

### 1.2. Sources of Oral Health Activities (OHA)-Educational Programmes and Interventions

Caring for children’s oral health is a shared moral duty among dental professionals and other professionals working with children, as well as parents and society [27]. Education for both parents and children can take place not only in dental clinics but also in paediatric and family medical centres. However, medical professionals often lack relevant training in oral health [28]. As a result, various oral health education programmes have been developed for health professionals who are not dental practitioners [27]. The reviewed literature underscores the need to improve the quality of education provided by experts in public health and oral health [29]. Numerous studies have shown that school education positively affects children’s oral health, knowledge, and behaviour [16,30]. Schools play a crucial role in developing healthy habits among young people. Educational programmes that use interactive learning methods, demonstrate proper toothbrushing techniques, and involve input from dentists have proven effective in improving oral hygiene and reducing caries among children and adolescents [14,18]. Numerous studies indicate that systematic education delivered by dentists to children and their parents through various programmes significantly decreases dental diseases in young people [31,32]. Some research has also examined the positive impact of peer education as a method (Peer-led oral health education model) [33] and interventions based on the theory of self-efficacy (Self-efficacy theory-based intervention) [34].

The use of the internet and social networks as a source of information is continually growing. Adolescents’ preferences for obtaining oral health information through social networks are becoming increasingly evident [35]. According to adolescents, smartphone app features related to oral health include disease detection, learning through games and rewards, fun, access to a dental practitioner, reminders, and ease of use [35]. Adolescents recognise the positive aspects of using smartphone applications for oral health and education; however, they are cautious about installing one [36]. Moreover, many studies question whether YouTube provides reliable information about oral health [37,38], while others find that videos can serve only as a helpful resource for parent education [29].

The authors of the present study therefore found a rationale for developing a new instrument (questionnaire) aiming to measure the sources of oral health activities (OHA), given the need to understand, and possibly to explain, current students’ OHA, as well as to determine the relation of these sources with already determined *OH types*. Knowledge on the OHA sources of university students could possibly be helpful in developing the university—based OHA educations or interventions. This study aimed to identify the sources of oral health activities (OHA) among university students by introducing an initial Oral Health Activities Sources Questionnaire (OHAQ-S), as well as to determine gender differences in OHA sources and the relationships between OHA sources and students’ oral health types (OH types).

## 2. Materials and Methods

### 2.1. A Sample of Respondents

The Raosoft Sample Size Calculator (Statistica 14.0) was used to determine the minimum required sample size. With an estimated university student population of 20,000 at the University of Split (Split, Croatia), for a confidence level of 99%, a margin of error of 5%, and a response distribution of approximately 50%, the minimum suitable sample size was calculated to be 643. A total of 658 students participated in the study, comprising 219 men (33.3%) and 439 women (66.7%). The mean age of the students was 21.33 ± 2.61 years, ranging from 18 to 26 years. Representation from various faculties and departments included: Faculty of Law (100 students, 15.2%); School of Dental Medicine (90 students; 13.7%); Faculty of Economics (90 students, 13.7%); School of Medicine (86 students, 13.1%); Faculty of Humanistic and Social Studies (78 students, 11.9%); Faculty of Electrical Engineering, Mechanical Engineering and Naval Architecture (75 students, 11.4%); Faculty of Kinesiology (53 students, 8.1%); Faculty of Science (46 students, 7%); and the University Department of Health Studies (40 students, 6.1%). The sample used in this study was identical to that in [39].

### 2.2. Sample of Variables

#### 2.2.1. Development of the Oral Health Activities Sources Questionnaire (OHAQ-S)

As part of a larger research project conducted at the University of Split, the Oral Health Activities Sources Questionnaire (OHAQ-S) was developed. The initial items for measuring students’ OHA sources were created in collaboration with three dental professionals, each of whom had 15 or more years of clinical experience. The variables in the OHA sources questionnaire were developed through two different methods. First, after the item selection process, three summary OHA source scales were formulated, each comprising multiple items. These scales were designed for sources of OHA that had been previously researched and validated in the scientific literature, as well as for the most common and potentially significant sources (parents, DMDs, and primary school sources) [16,18,19,30]. Second, the remaining measurements associated with OHA sources were single-item constructs, each defined by a single item aimed at measuring the respondent’s attitude towards OHA sources. They were developed as single items because dental experts estimated that these could be significant OHA sources for students. For each of the remaining and potential OHA sources (such as kindergarten, high school, self-learning, friends, media & internet, and university sources), two single-item variables were created and used in further analyses. Due to the brevity and efficiency of the constructed questionnaire, it was not designed as a summary scale (i.e., an operational functionalization of an existing hypothetical construct), unlike the previous three measures of general and “primary” sources. By using two different items for each possible OHA source, the experts aimed to cover a broader range of source measurements. Each questionnaire item was evaluated using a five-point Likert scale (1—completely false; 2—mostly false; 3—partially true; 4—mostly true; 5—completely true).

#### 2.2.2. OHAQ Variables

In 2022, Aranza et al. [39] developed the Oral Health Activities Questionnaire (OHAQ), which assesses various activities and behaviours related to the oral hygiene routines of university students, using five measures: the basic oral hygiene activities (BOHA) scale, the orientation to a doctor of dental medicine (DMDO) scale, the regularity of tooth brushing (ROTB) scale, the use of dental floss (FLOSS) scale, and the overall OHAQ score (OHAQ SUM). Female and male students showed differences on the OHAQ measures, with females achieving higher, more desirable results than males across all measures: the four OHAQ scales and the overall score. Based on the OHAQ results, Aranza et al. (2022) identified four oral health types (OH types) for both female and male students: excellent, good, satisfactory, and poor. In the present study, five OHAQ questionnaire variables (OHAQ scales and overall score results) and the oral health type membership variable were used [39].

### 2.3. Data Collection Procedure

This study was conducted in full accordance with the World Medical Association Declaration of Helsinki and approved by the Ethics Committee of the University of Split, University Department of Health Studies (No. 2181-228-06-14-0002, 31 March 2014). The authors contacted the faculties of the University of Split to recruit potential participants. Students at the University of Split were invited to participate in this study at the beginning of the summer semester. The questionnaire was administered in regular class groups, and students were asked by their faculty to remain in class at the end of a lecture to participate voluntarily. The aims and objectives of the study were explained to the students, and they were instructed how to complete the questionnaire. All students were asked to complete the OHAQ questionnaires (OHAQ and OHAQ_S) anonymously after signing a separate consent form for participation. The questionnaire covered more variables than those in the present study, and the students completed it in 15–20 min. A tiny number of questionnaires (less than 1% of the overall sample) were excluded from the analyses at the end of the study, mainly because participants did not answer, or answered unclearly, as to several items.

### 2.4. Data Processing Procedures

To assess the *homogeneity* of the scales within the OHA sources questionnaire, an exploratory Principal Component Analysis (PCA) with Varimax rotation was employed, using the Guttman–Kaiser criterion to determine the number of significant components. To evaluate the *reliability* of scales, Cronbach’s alpha coefficient was calculated. The *sensitivity* of all measures in the OHA sources questionnaire was assessed using the Kolmogorov–Smirnov *goodness-of-fit test*, the range of results (minimum and maximum), and *skewness* and *kurtosis* indices. The results for the scales of the developed OHAQ-S questionnaire were obtained by summing all item scores within each scale, then dividing this total by the number of items (sum/number of items). This approach allowed for easy comparison of the results’ expressiveness across different measures, including scales with varying item counts and single-item variables within the OHAQ-S questionnaire. Descriptive statistics for all measures of the OHAQ and OHAQ-S questionnaires were presented as mean, standard deviation, minimum, and maximum values. All results were interpreted according to the guidelines [40]: *extremely low* (1.00–1.99), *very low* (2.00–2.39), *low* (2.40–2.79), *moderate* (2.80–3.20), *high* (3.21–3.60), *very high* (3.61–4.00), and *extremely high* (4.01–5.00). For the purpose of initial validation of the OHAQ-S questionnaire measures, the following forms of *construct validity* were determined: *discriminant validity* (determining possible differences between groups of respondents within the sample) and *predictive validity* (determining the predictive relationship of OHAQ-S measures with a dependent, criterion variable). Gender differences in OHAQ-S measures were analysed using Student’s *t*-test for independent samples. The influence and determinants of OHA sources on the overall OHAQ score were examined in both female and male subsamples using hierarchical multiple regression analysis (HMRA). Differences in OHAQ-S measures between subgroups based on OH type, separated for female and male participants, were analysed in two ways: first, by applying univariate one-way analysis of variance (One-way ANOVA) along with the effect size measure *eta^2^* (*ƞ^2^*) and *post hoc* Fisher’s *least significant difference* (LSD) test for detailed comparison; second, through conducting multivariate discriminant analysis.

## 3. Results

All constructed measures of the new *Oral Health Activities Sources Questionnaire (OHAQ-S)* are presented, including scales (with associated indicators of metric characteristics such as *homogeneity* and *reliability*) and single-item variables (Table 1).

In the OHAQ-S questionnaire, three OHA source scales were developed with acceptable metric properties of *homogeneity* and *reliability*. In each scale, all items projected only one principal component (scale *homogeneity*). Although the *reliability* of the two scales falls below the commonly accepted internal consistency coefficient of 0.70, their reliability can still be considered *conditionally satisfactory* because these were newly developed scales intended for pilot research purposes, and the associated results do not directly influence decision-making regarding the respondents completing the OHAQ-S questionnaire [41]. The *parent source* scale comprises four items and assesses parents’ oral hygiene instruction given to their children. The most significant and frequently mentioned item (3.42, high) in this scale was “*My parents demonstrated brushing my teeth.*” The *DMD source* scale comprises five items focusing on educational activities for DMD children and youth related to oral hygiene. The most important and highest-rated item (2.83, moderate) in this scale was “*the dentist explained to me the rules for maintaining oral hygiene*.” The *primary school* source scale comprises four items related to oral hygiene education activities conducted during primary education. The most notable and highest-rated item (3.04, moderate) in this scale was “*I learned how to maintain dental and oral hygiene in primary school*.”

The remaining OHA source measures were single-item constructs, in which variables were defined by the content of individual items used to assess behaviour and/or attitude. The average scores for these single-item variables range from very high (3.66) for the item ‘*I learned how to brush my teeth in kindergarten*’ to extremely low (1.69) for the item ‘*I gained the knowledge necessary to maintain oral hygiene at university*.’

A detailed overview of the OHAQ-S questionnaire results is provided in Table 2, including the calculation of gender differences. The variables were organised in an assumed “chronological” sequence. The earliest potential OHA sources, such as *parents* and *kindergarten sources*, were shown first, followed by *childhood* and *early youth sources* (*primary school*, *DMD*, and *high school sources*), and finally the remaining, potentially later OHA sources (*self-learning*, *media & internet*, *friends*, and *university sources*) were shown.

For both subsamples, as to female and male students, the *sensitivity* indicators revealed different findings. The Kolmogorov–Smirnov *goodness-of-fit* test was expectedly significant for most OHAQ-S questionnaire measures, given its known high sensitivity in large samples. However, in both subsamples, only one measure (female student *university source—acquired knowledge)* had *skewness* and *kurtosis* indices exceeding ±2.00, which the authors of [42] state are almost always acceptable for assessing a *satisfactory* level of measure *sensitivity*. The mentioned measure was negatively asymmetric due to the very high number of lowest-possible-item estimates in the female subsample. Considering the *sensitivity* indicators of the OHAQ-S measures, the authors concluded that continued use of parametric statistical indicators and procedures in subsequent analyses was justified.

*Gender differences* were observed in six OHAQ-S questionnaire variables across five distinct “secondary” OHA sources: *university*, *high school*, *self-learning*, *friends*, and *kindergarten*. Female students scored *higher* than male students on variables related to *self-learning* and *kindergarten*, while scoring *lower* on those related to *university*, *high school*, and *friends*. No gender differences were found for the three “primary” OHA sources measured by scales: *parents*, *DMD*, and *primary school* sources. Since gender differences appeared in multiple OHA sources—and noting that gender differences were also detected in all variables of the OHAQ questionnaire assessing oral hygiene activities (variables BOHA, ROTB, DMDO, FLOSS, and OHAQ overall score) [39]—the interpretation of the average OHAQ-S results and subsequent statistical analyses were conducted separately for female and male subsamples. Female students rated the primary OHA sources as *moderate* (*parents* source) or *low* (*DMD* and *primary school* sources). For secondary sources, they rated *kindergarten* source as *very high*, *self-learning* source as *high*, *internet* source as *low*, and *high school, media, friends*, and *university* sources as *very low* or *extremely low*. Male students evaluated primary OHA sources as *moderate* (*parents* source) or *low* (*DMD* and *primary school* sources). Regarding secondary sources, they rated *kindergarten* source as *high*, *self-learning* source as *moderate*, the *internet* source as *low*, and *high school*, *media, friends*, and *university* sources as *very low* or *extremely low*.

Groups of predictor variables were incorporated into the HMRA analysis following the authors’ “chronological” assumptions and order regarding OHA sources. Those predictor variables representing possible *early childhood* OHA sources were included in the regression analysis at step 1 (Model 1). Possible *childhood*, *early* and *middle adolescence* OHA sources were included at step two (Model 2), and all *other potential OHA sources* were added at step three of the analysis (Model 3).

The results of the HMRA analysis among female students showed that all three regression models were significant, with OHA sources explaining 7.3%, 19.9%, and 30.2% of the variance in the overall OHAQ score. In HRMA Model 1, the *parent* source was the only significant predictor. In Model 2, the *DMD source* was the most significant predictor, with *parents*, *kindergarten (related to early tooth brushing practice)*, and *primary school* sources as additional significant predictors of the student’s overall OHA activities. In the HRMA Model 3, DMD, *self-learning (related to dental floss use)*, and *university (related to oral hygiene knowledge)* sources were the most significant predictors, with *friends*, *parents*, *kindergarten*, and *high school* sources additionally contributing to the prediction of the student’s overall OHA activities. In Model 3, the predictive effect of the *primary school* source was no longer significant, whereas the *high school* source emerged as significant. *Early childhood* sources explained 7.3% of the variance, *childhood and early adolescence* sources explained an additional 12.5%, and *other and later adolescence* sources explained an additional 12.7% of the variance in the female students’ overall OHA activities. Overall, the predictors explained 30.2% of the variance in the dependent variable. Multicollinearity among predictors was not detected (all tolerance coefficients were above 0.10).

The results of the HMRA analysis among male students revealed that all three regression models were significant, and that OHA sources explained 11.6%, 22.9%, and 34.1% of the variance in the OHAQ overall score. In HRMA Model 1, the *parents* source was the only significant predictor. In Model 2, the *DMD source* was the most significant predictor, and the *parents* source was also a significant predictor of the student’s overall OH activities. In HRMA Model 3, the *DMD* and *university sources (related to oral hygiene knowledge)* were the most significant predictors, with *parents and self-learning* (*about dental floss usage*) sources, and *friends* source (*about dental floss usage*) as additional significant predictors of the student’s overall OHA. The *primary school* source was not a significant predictor in any model, although the *high school* source was very close to significance (*p* = 0.06). *Early childhood* sources explained 11.6% of the variance, *childhood and early adolescence* sources explained an additional 11.4%, and *other and later adolescence sources* explained an additional 11.1% of the variance in the male student’s overall OHA. Overall, the predictors accounted for 34.1% of the variance in the dependent variable. Multicollinearity among predictors was not detected (all tolerance coefficients were above 0.10).

Aranza et al. (2022) found gender differences on the OHAQ measures, and identified four characteristics *OH types* based on the OHAQ questionnaire measures separately for subsamples of female and male students [39]. In both subsamples, separated by respondents’ gender, OH types have the same names, but their frequencies and the expression of OHAQ measures differ. After determining the determinants of the OHAQ overall score in Table 3 and Table 4, analyses of differences in OHAQ-S measures (OHA sources) by the variable of *OH type membership* were conducted for each subsample of female and male students: univariate analyses (*One-way ANOVA*) shown in Table 5 and Table 6, as well as multivariate analyses (*discriminant analysis*), as shown in Table 7 and Table 8.

The ANOVA results show that the *OH types* groups of female students differ significantly across 7 of 15 OHAQ-S variables. Using Fisher’s LSD *post hoc* test, differences between groups were found in two additional OHAQ-S variables, with these differences being very close to the threshold of statistical significance (*p* < 0.10). The most notable differences among OH types were seen in the OH source variables: *DMD source*, *self-learning (dental floss usage)*, *university*, *friends*, and *parents* sources. According to Cohen’s (1988) [43] criterion for differences-effect size, as measured by the eta-squared coefficient, of the nine identified differences, only two—*DMD source* and *self-learning (dental floss usage)* sources—can be classified as having a *medium* effect size.

The ANOVA results show that the *OH types* of male students differ significantly in 10 out of 15 possible OHAQ-S variables. Using Fisher’s LSD *post hoc* test, there were two additional variables where the differences were very close to the threshold of statistical significance (*p* < 0.10). The most notable differences among OH types were observed in the following OH source variables: *DMD source, parents source*, *high school, university*, *self-learning (dental floss usage)*, *media & internet (health journals)*, and *friends* *sources*. According to the criterion for differences-effect size (Cohen, 1988) [43], as measured by the eta squared coefficient, only one of the 12 identified differences can be classified as a *significant* and *large* effect size (*DMD source*), while eight of the differences were classified as *medium* effect sizes (most notably in *university*, *self-learning*, *parents,* and *media & internet* sources).

Since many significant differences in OHAQ-S measures between student subgroups based on OH type were observed in both subsamples by gender, and because several significant intercorrelations among OHAQ-S measures were identified (which was expected, since two single-item measures were used for six OHAQ-S sources), multivariate *discriminant analysis* was conducted, and the results are presented in Table 7 and Table 8.

Discriminant analysis identified two significant discriminant functions that differentiate OH types among female students based on their OHAQ-S sources. The first discriminant function (DF) separated students with *excellent OH* activity from all other groups, especially those with *poor OH* activity. The sources that most differentiate *excellent OH* female students were *higher* levels of *self-learning—dental floss usage* (0.59) and *DMD* sources (0.55)—and there were *slightly higher* contributions from *university—acquired OH knowledge* (0.34), *university—fully educated* (0.26), and *parent* (0.30) sources. Consequently, female students with higher-quality and higher OH type (*excellent OH type*) tend to gather significantly more OH activities from the *DMD* and *self-learning—about dental floss* sources, and information from the *university* and *parents* sources than those with lower OH types, especially when compared with the *poor OH type* group.

The second discriminant function (DF) primarily distinguished female students with a *satisfactory OH type* from those with other types, especially those with *good OH type*. The OH sources that most differentiate the *satisfactory OH type* of female students from the *good OH type* were *higher-*expressed sources: *friends—the best information* (0.56) and *self-learning—dental floss* (0.51), followed by *slightly higher*-expressed sources *media & internet* (0.29) and *high school—then I learned the most* (0.27), and finally the *slightly lower*-expressed sources *kindergarten—I learned to brush my teeth* (0.28) and *self-learning—I learned the most by myself* (0.25). Therefore, those students belonging to a higher-quality OH type (*good OH type*) acquired significantly fewer OHA in the sources of *friends*, *self-learning—about dental floss*, *media & internet* than those with a lower OH type (*satisfactory OH type*).

The discriminant analysis also identified two significant discriminant functions that differentiate the OH types of male students based on their OHA sources. The first discriminant function (DF) separated the group of male students of the *excellent OH type* from all other types, and mainly from the *poor OH type*. The OH sources that distinguish the *excellent OH type* of male students from other types were significantly *more prominent*: *DMD source* (0.66), *self-learning source—dental floss* (0.53), *university source—acquired OH knowledge* (0.48), *parents source* (0.46), and *media & internet source—health magazines* (0.45).

The second discriminant function (DF) most clearly separated male students with *excellent OH type* from those with *satisfactory OH type*. The OH sources that most differentiate the *satisfactory OH type* of male students from the *excellent OH type* were as follows: first, more prominent *friends sources—about dental floss* (0.53), *high school source—then I have just learned that* (0.42), *university sources* (0.33, 0.37), *media & internet source—health magazines* (0.35); second, *less prominent* early sources, such as *parents source* (0.30) and *kindergarten sources* (0.32, 0.31).

## 4. Discussion

The most important findings of this study were as follows: The Oral Health Activities Sources Questionnaire (OHAQ-S) was developed to measure *primary OHA sources (parents, DMD, and primary schools)* and secondary OHA sources with satisfactory measurement characteristics. No *gender differences* between female and male students were found in the primary OHA sources. However, *gender differences* were identified in several secondary OHA sources (*university, high school, self-learning, friends,* and *kindergarten* sources). Female students have different OHA sources than male students, and a greater reliance on the *self-learning* source than found among male students;

- Thedeterminants of the overall OH activities for female students were primarily two primary sources (*parents* and *DMD sources*) and several secondary sources (*self-learning—dental floss usage, university—acquired OH knowledge, friends—importance of dental floss, kindergarten—I learned to brush my teeth, high school—I learned the most then*).
- In the subsample of female students, several significant differences in OHAQ-S sources were identified among the four *OH types*. The *excellent OH type* of female students differs most from the other types in terms of *higher*-expressed sources: *self-learning, dental floss usage,* and *DMD sources,* and the *slightly higher*-expressed *university sources* and *parents source*.
- In the subsample of male students, many significant differences in OHAQ-S sources were observed among the four OH types. The *excellent OH type* of male students differs most from other types by having *higher*-expressed sources *DMD source, self-learning—dental floss usage source, university source—acquired OH knowledge, parents source*, and *media & internet source—health journals*.
- Considering the sources in OHAQ-S, several similarities and differences in determinants of OHA between female and male students were identified. All these findings are discussed in detail in the subsequent sections of the discussion.

### 4.1. Gender Differences of Student’s OHA Sources

Three scales of OHA sources with satisfactory metric characteristics were developed within the OHAQ-S questionnaire. These three scales refer to the most significant and *primary* OHA sources [8,14,18,19,20], and these OHA sources for students include the *parent source, the DMD source,* and *the primary school source*. The *parents source* involves parental teaching, practice, and supervision of their children’s oral hygiene, representing the earliest source of OH activities [20]. The *DMD source* pertains to the advisory and educational activities on oral hygiene conducted by a DMD during regular check-ups and/or clinical interventions with children and adolescents [13]. The *primary school* source refers to oral hygiene education activities carried out during children’s primary education. The influence of parents on children’s oral health has been confirmed in many studies [20], as has the positive impact of school-based education on children’s oral health, knowledge, and behaviour [16,30]. An RCT conducted in Iran [44] demonstrated that school-based and kindergarten-based interventions grounded in learning theories, such as social cognitive theory, led to substantial improvements in knowledge, independence, and oral habits through educational games, posters, digital groups, and leaflets. The results showed a +48.5% increase in twice-daily tooth brushing, a +64.2% increase in flossing, and a 38% reduction in plaque. Moreover, the dental practitioners’ role significantly contributes to improved oral hygiene habits among young people [15]. The remaining single-item measures of OHA sources (*high school*, *kindergarten*, *self-learning*, *media & internet*, *friends*, and *university* sources) represent potential sources that were likely linked to the motives (interests and/or needs) or the educational programme of young people during their upbringing and development.

For the three “primary” OHA sources (parents, DMD, and elementary school), no *gender differences* were observed. Therefore, the expression of these three, the most significant and primary sources, was independent of the respondent’s gender. All observed gender differences between female and male students were detected by the “secondary”, single-item OHA sources: *university*, *high school*, *self-learning*, *friends*, and *kindergarten* sources. Female students score *higher* than male students in the variables of *self-learning* and *kindergarten* sources.

Male students have higher scores than female students in the variables of *university, high school*, and *friends* sources. The gender differences identified by various “secondary” sources are briefly listed in the following: source *I learned the most about OH in high school*, male students have *very low* scores compared to *extremely low* for female students; source *I gained the knowledge about OH at university*, although the scores of both subgroups were *extremely low*, male students still have *higher* scores than female students; source *I learned about OH by myself*, female students have *moderate* scores compared to *low* scores for the male students; source *my friends gave me the best information about OH*, male students have *very low* scores compared to *extremely low* for female students; source at university *I was fully educated about the importance of OH*, male students have very low scores compared to extremely low for female students; and, source kindergarten *I remember the story about the Tooth Fairy*, female students have *extremely high* scores compared to *very high* for male students.

Considering the findings on gender differences, it was possible to conclude that female and male students have different OHA sources, with male students acquiring their OHA from more sources than female students. Furthermore, given the gender differences in OHAQ-S sources identified here and OHAQ activities [39], it was appropriate to analyse the findings for each gender subgroup separately and then compare and interpret them only descriptively.

### 4.2. OHAQ-S Sources as Determinants of Students’ Overall OHA Behaviour

When assessing OHA sources as potential determinants of total OHA within the subsample of female students, several key findings emerged: OHA sources across all three regression models (introduced in sequence: *early*, *middle*, and *other* sources) were significant determinants of students’ *overall OHA* behaviour measure, but the overall *effect-size* of the regression was *modest*; “*later” OH sources* (*middle* and *other* sources) have a greater impact on total OHA than *early sources* (*parents and kindergarten sources*); in the regression model of *early OH sources* (Model 1), the *parents source* was the only significant determinant; in the regression model of *childhood*, *early and middle adolescence OH sources* (Model 2), all three *primary OH sources*—*parents*, *DMD*, *and primary school sources*—were significant determinants of total OHA, including the *kindergarten source—I learned to brush my teeth*; in the general regression model (Model 3), among the *early and middle OH sources*, two primary sources (*parents and DMD sources*) and two secondary sources (*kindergarten—I learned to brush my teeth,* and *high school—then I learned the most*) remained significant determinants. These determinants were complemented by several *other* sources of *self-learning—dental floss, university—acquired OH knowledge*, and *friends—importance of dental floss*. The significance of *primary school* source as a determinant of OHA activity ceased with the entry of these “other” determinants; early OHA sources contribute less to explaining overall OHA activities than do middle and other OHA sources.

When analysing OHA sources as potential determinants of overall OHA within the subsample of male students, several key findings emerged: OHA sources were significant determinants of the *overall OH activities* measure among students across all three regression models (introduced as *early*, *middle*, and *other* sources), but the overall *effect-size* of the regression was *modest*; *early*, *middle*, *and other OH sources* contribute roughly equally to determining the *overall OHA*; in the regression model for *early OH sources* (Model 1), only the *parents source* was a significant determinant; in the regression model of *childhood*, *early and middle adolescence OH sources* (Model 2), only two primary OH Sources—*parents and DMD sources*—were important determinants of overall OHA, and the *primary school source* was not significant; in the general regression model (Model 3) two primary sources—*parents and DMD* sources—were “confirmed” and represented again as significant determinants, but with three associated “other and later” secondary sources: *university—acquired OH knowledge*, *self-learning—dental floss, and friends—importance of dental floss*.

The findings of this study partially confirm some previous scientific results. A systematic review and meta-analysis of school-based oral health education interventions on oral health status and oral hygiene habits in schoolchildren showed a beneficial impact on several outcomes, such as reduced plaque and gingivitis, improved knowledge, attitude, and behaviour, and better oral hygiene [45]. Child age and frequency of dental check-ups were positively associated with dental caries prevalence [46] and parental oral health literacy predicts children’s oral health outcomes [47].

When comparing the established determinants of *overall OHA* across the set of OHAQ-S sources for both gender subsamples (female and male students), several similarities and differences emerged. The following were the common and similar findings on determinants of *overall OHA* between the two gender subgroups: first, two *primary sources (DMD source* and *parent source)* were consistently significant determinants of OHA; second, three *secondary sources* of OHA were the same (*university—acquired OH knowledge, self-learning—dental floss,* and *friends—importance of dental floss*); third, the *primary school source* in the general regression model (Model 3) was not a significant determinant of *overall OHA*; and fourth, *media & internet sources* were not significant determinants of *overall OHA* in any of regression models.

The differences in the findings regarding the determinants of *overall OHA* between the two gender subsamples were as follows: first, it was evident that female students have a *higher* number of earlier OHAQ-S sources compared to male students (*kindergarten source—I learned to brush my teeth* and *primary school source* were also significant determinants for them); second, in general, the structure of OHAQ-S determinants of total *overall OHA* varied because for female students sources *high school—I learned the most* and *kindergarten—I learned to brush my teeth* were determinants of their *overall OHA*.

These findings also partially confirm some previous scientific results. A comparison of oral health behaviour between men and women in the USA [48] showed that men tended to visit the dentist less often, had poorer perceptions of gum and tooth health, had poorer flossing habits, and experienced more root caries. Women were more proactive in seeking dental care and showed greater awareness of oral health. Furthermore, a review [49] indicated that men also visit dentists less frequently and, compared to women, seek oral treatment more often for acute problems and less for disease prevention. Women display more positive attitudes towards dental visits, are more knowledgeable about oral hygiene, and maintain better oral hygiene habits than men.

### 4.3. Relationships Between OHAQ-S Sources and Types of OH

Aranza et al. (2022) developed typologies of both female and male students based on their expression of OH activities [39]. Although four different types of female and male students share the same names, gender differences were significant, both in the expressiveness of individual activity measures in the OHAQ questionnaire and in the relative frequency of members of each type within the gender subsample. An analysis of differences in OHAQ-S sources between groups of students affiliated with different OHA types revealed numerous disparities. Univariate analyses of both female and male student subsamples showed many differences in OHAQ-S sources across OHA types.

In the subsample of female students, several key findings were identified univariately: the *excellent OH type* was characterised by *more prominent* OHAQ-s sources than other *OH types* (*DMD source*, *self-learning source—dental floss usage*, *university sources; kindergarten source—I learned to brush my teeth*) and the *lowest* levels of *media & internet sources*; the *good OH type* differs from the *satisfactory OH type* by having *lower* expression of these sources: *self-learning source—dental floss usage*, *media & internet sources (both)*, *and friend sources–best information*, and the *poor OH type* was marked by the *lowest* expression of the *DMD source*, *self-learning source—dental floss usage*, *and university source—acquired OH knowledge*. *Poor OH type* also shows decreased expression of the *parent and kindergarten sources—I learned to brush my teeth*, and a *higher* expression of the *friends source—best information*.

Furthermore, in the female student’s sub-sample, several significant findings were identified through multivariate analysis: female students of the *excellent OH type* differ from all other types and mostly from those with *poor type* of OH activities. The OHAQ-S sources that most distinguish the *excellent OH type* of female students from other types were the *DMD source*, the *self-learning source—dental floss usage*, the *parents source*, and the *university sources*; female students with *satisfactory OH type activities* differ from other types, especially those with *good OH type*. The OHAQ-S sources through which the *satisfactory OH type* of female students differ most from the *good OH type* include “later” sources: *friends—best information, self-learning—dental floss, media & internet*, and *high school—I learned the most*, *self-learning—I learned most myself*, as well as earlier source *kindergarten—I learned to brush my teeth*. Based on the aforementioned manifest and univariate findings, as well as on the multivariate results, it is possible to claim that those analyses provide additional insight into the sources of OHA, and into the influencing and shaping of *overall OHA behaviour*. As well, it could be claimed that considering findings solely at the level of the entire subsample of female students is somewhat inappropriate. Specifically, the *excellent OH type* of female students (about 20% of the sample) was characterised by OHA mainly based on communication and education relative to a *DMD doctor*, and subsequently through learning during *university studies*, through *self-learning—about using of dental floss*, and with limited use of *media & internet sources*. These subjects could primarily relate to solid, reliable sources of knowledge about OHA (*DMD and university sources*), have high levels of “experience” with *learning to brush their teeth* in the kindergarten, and have had very little relationship with popular and unreliable *media & internet sources*. Female students who belong to the *good OH type* (approximately 27%) reported slightly fewer OHA from sources such as *friends*, *self-learning about dental floss*, and the *media & internet* than those with a very similar *satisfactory OH type*. *Satisfactory OH type* (around 27%) female students rely on less reliable sources such as *friends*, *media*, *internet*, and *self-learning—dental floss.* In contrast, *poor OH type* female students (about 26%) rely the least on *DMD and university sources*, “miss” early experiences from *parents and kindergarten sources*, and were related more to the emotionally close and highly “unreliable” *friends source—best OH information*. Overall, female students with higher OHA levels mostly have sources such as *DMD (doctor of DM), self-learning—dental floss use*, *university sources,* and *parent sources*.

In the male students’ subgroup, several significant findings were identified univariately: the *excellent OH type* was characterised by the *most prominent* OHAQ-s sources relative to all other OH types: *DMD source*, *parent source*, *kindergarten source—I learned to brush my teeth*, and *self-learning source—dental floss usage*; *good OH type* differs from *satisfactory OH type* in that it has *lower*-expressed sources: *DMD source, primary school source*, *high school sources (both), media & internet source—health journals*, *friend sources (both)*, *university sources (both)*, and *primary school source*, but also a *higher*-expressed *kindergarten source—I learned to my brush teeth.* The lowest levels of specific OHA sources characterise the *poor OH type*: *DMD source, parent source*, *self-learning source—dental floss usage*; *media & internet source—health journals;* and *university source—gained OH knowledge*. It also shows *lower* levels of *kindergarten source activities—I learned to brush my teeth*; *high school sources (both)*; and *friend source—best information.*

Furthermore, a few key findings were established through multivariate analysis in the male students’ subgroup. Similar to women, male students with an *excellent level of OHA* differ from all other types, mainly from the *poor OHA type*. The OHAQ-S sources that most distinguish the *excellent OH type* of male students from others were the *more* prominent *DMD source*, *the parents source*, *the self-learning source (dental floss usage)*, *the university sources*, and *the media & internet source*. Male students with a *satisfactory level of OH activities* differ from those with an *excellent OH type*. The OHAQ-S sources that mainly distinguish the *satisfactory OH type* of male students from the *excellent OH type* was the *higher*-expressed source for *friends—dental floss usage* and *media & internet*, and *lower*-expressed *DMD and parents sources*. Like the female student’s sub-sample, these multivariate analysis results enhanced our understanding of the sources of OHA and *OH types* among male students. The *excellent OH type* of male students (about 10% of the sample) was characterised by OH activities mainly centred on communication and education provided by the *DMD doctor and parents*, as well as on *self-learning—dental floss using.* They mainly rely on traditional sources (*DMD and parents sources*) for knowledge about OHA. Male students with a *good OH type* (around 32%) demonstrate very high *regularity in tooth brushing* but extremely rare *dental floss usage* score. *Satisfactory OH type* (about 30%) male students had a closer relation to *university sources* than to “less reliable” sources (*friends*, *media & internet*, *self-learning—dental floss,* and *high school sources*). *Poor OH type* (around 28%) male students had least relation to *DMD and university sources* and they “miss” OH experiences from the *parents source*. As with female students, male students with a higher type (and higher level) of OHA mostly were related on *DMD source, parent source*, and *self-learning—dental floss usage*, and they had more experience from *kindergarten—I learned to brush my teeth*.

Although female and male students differ in their *overall OH activity* levels [39], the findings regarding the sources of OHAQ-S across different OH types were very similar. For instance, the sources linked to *excellent OH types* were fairly similar (*DMD*, *parents*, *university*, and *self-learning—dental floss use* sources). However, around 20% of female respondents were classified as having an *excellent OH type*, compared to about 10% of male respondents. Similar patterns were observed for the other OH types within female and male students’ subsamples.

### 4.4. Possible Implications and Recommended Practices

Based on the results obtained regarding differences in the sources of oral hygiene activities among various *OH types* of female and male students, it can be concluded that these findings contribute to the understanding of students’ *OH activities types* and patterns, as identified by Aranza et al. (2022) [39]. These results also provide a valuable information basis for possible planning and implementing practical activities for targeted oral hygiene education for university students.

As well, the findings emphasise the need for a systematic, gender-specific approach to oral hygiene education. Based on a literature review and partially on the present study results, we make the following recommendations: to consistently motivate parents and educational institutions, particularly kindergartens, to promote good oral hygiene habits from a young age; to prepare dentists capable of playing key roles as educators, advisors, and supervisors in guiding young people towards proper oral hygiene practices; to ensure high-quality education provision—as to both planners and implementers of programmes—in primary and secondary schools, involving experts from public health institutions invited to conduct regular examinations and deliver targeted education (in Croatia, these could include the *Teaching Institute for Public Health* or other specialised groups). Special attention should be given to implementing educational interventions during the early adolescent years (between 11–12 and 15–16 years), when enduring attitudes and patterns of oral hygiene behaviour begin to develop. Timely, targeted, and effective education during this period could substantially help maintain and improve young people’s oral health in the long term.

At the university level, it is advisable to support the development of specialised educational programmes on oral hygiene, since students generally express a willingness and need for additional knowledge. Furthermore, media outlets—both print and electronic—as well as online platforms, can play a significant role, provided that the information and education on OH topics are grounded in scientific evidence. Incorporating models of exemplary OH behaviour—such as well-known public figures, musicians, actors, dancers, athletes, young web influencers, or other “role models” for youth—can also aid in conveying positive messages in an accessible and motivating manner (i.e., not only educating through lecturing and teaching “from above” by experts, but also through personal examples and through “youthful” acceptance and understanding).

Considering the findings of this study within the broader context of previous scientific research, it is possible to state that earlier studies show that a combination of formal educational programmes in schools and kindergartens, support from dental professionals, involvement of parents, and the use of digital tools (platforms) play a key role in promoting children’s oral health. Even brief kindergarten education, grounded in learning theories and employing interactive methods (e.g., games, animations, and tooth models), can significantly enhance children’s knowledge and habits [44,50,51,52]. A key role in educating university students about proper oral hygiene could be played by a combination of professional support, practical workshops, and digital educational content that raises awareness of the importance of daily oral health care. Such “mixed” approaches can effectively motivate young people to adopt healthy habits and help maintain their overall health in the long term.

In conclusion, based on this study and previous scientific findings, the authors believe it is justified to recommend guidelines for designing oral health (OH) education for young people:

- We recommend the early (but also later) implementation of OH educational programmes using multimodal and interactive teaching methods and procedures.
- Where possible, one should aim to educate parents about OH beforehand or to involve parents in the already established education of children and young people by presenting the parents with the content shown and adopted by the children (as this could preserve or even enhance the effects of the prior education with children).
- We recommend involving dentists (DMD) in all school-based education, both in partially modelling the planned curriculum based on prior clinical practice experience and directly delivering some of the teaching topics.
- It is advisable to introduce basic health and media literacy education within schools, focusing on decision-making in health activities (e.g., about Informed Health Choices) with respect to young people. This can include education similar to that conducted with students in grades 3 and 6 of primary schools. Such education can help young people make better, more effective decisions about general health and oral health (OH)-related activities [53].
- We recommend the creation or adaptation of various digital tools or programmes that could help young people in conducting a comprehensive self-assessment of their oral health (OH). These tools could also guide them in carrying out recommended OH practices or direct them to additional “educational” activities, such as viewing specialised webinars tailored to their needs or preferences. The authors of this study consider that university students can complete the OHAQ questionnaire [39] online to assess their overall oral health. Based on their results (*overall OHAQ score* and the scores for its four components), they can be encouraged to participate in the suggested activities designed to maintain OH. These activities could include watching brief, focused webinars or booking an appointment with a university dentist for preventive, educational, or clinical treatment.

### 4.5. Study Limitations

The present study has several limitations that may affect the generalisability of the findings. The study used a *cross-sectional design*, which does not allow for causal conclusions. Specifically, this research design could not capture all potential interactions, influences, and interdependencies among different sources of OH in real time, and could only capture their partial and collective contributions at the moment of measurement (when respondents were already involved in university studies). Absence of longitudinal data limits the possibility of establishing the causal relationships between OHA sources and other measures.

University students answered the questionnaire items related to previous OHA sources from memory at the time of filling out the OHAQ-S questionnaire, so it is possible that the research results were influenced by the phenomenon of *recall bias*. It is reasonable to assume that the time gap from the moment of the possible influence of a particular OHA source led to *cognitive* and *information distortion*, in which students do not accurately remember previous experiences or influences. In any case, possible *recall bias* might have a negative influence on the validity of questionnaire based on and collecting self-reported data. *Recall bias* could influence the validity by distorting associations between variables or by leading researchers to incorrect conclusions.

This study also has many limitations related to the quality of the applied or omitted *psychometric procedures* that are usually carried out in the construction and validation of new questionnaires. In this study, the lack of an external validation procedure for the OHAQ-S questionnaire measures was obvious: the measurements of the new constructs were not compared with any of the existing measurement instruments for measuring OHA sources. Second, part of the *initial validation* (analysis of differences in OHA source measures between groups of different OH types) was also carried out on self-reported measures of the OHAQ questionnaire. Third, since we had only one contact with university students, during which they anonymously filled out the questionnaire, no test–retest reliability measurement of the OHAQ-S questionnaire was carried out.

Single-item measures of the OH sources were undoubtedly of lower measurement quality compared to the constructed summary scales in the OHAQ-S questionnaire. However, they were utilised in this study for several reasons: first, the measurement of OH sources was neither the sole nor the principal aim of the broader OH measurement project (and related measures, such as the OH sources in this study) involving university students; second, due to the targeted (necessary) “economy” involved in measuring students’ OH, there was insufficient “space” to develop numerous scales of OH sources within the comprehensive questionnaire used in the project; third, we were most interested in potential primary OH sources such as parents, primary school and DMD sources, for which multiple items were used, resulting in the creation of summary scales as multiple operationalised constructs of OHA sources.

For the purpose of implementing HMRA, the OH source measures from the OHAQ-S questionnaire were, by the authors of this study, divided into three determinant models in an assumed “chronological” order. However, the actual chronological sequence of the influences and effects of OH sources may differ somewhat. Some OHAQ-S sources were nonetheless clearly linked to chronological age and were therefore determined chronologically (e.g., kindergarten, primary school, secondary school, and university).

The broader cultural, institutional, educational and healthcare system specific to the Croatian university environment, as well as the general Croatian society in which the study was conducted, must not be overlooked. Thus, the findings and conclusions derived from this study cannot simply be generalized and extrapolated to other cultural and/or educational environments.

Finally, all of these previously mentioned limitations may have important negative implications for the validity of the conclusions yielded from the study results. Conclusions made based on the potentially weak and unreliable results should be considered cautiously and carefully keeping in mind these possible weaknesses and shortcomings.

### 4.6. Future Research Directions

Future research may partially be determined by the previously mentioned limitations of the present study. However, the authors also have a broader perspective on potential further studies related to the OHAQ-S source measures. First, it is recommended that researchers conduct more cross-sectional measurements of OHA sources among respondents from younger age groups: late-middle childhood (around 10–11 years), early adolescence (around 13–15 years, final primary school grades), and middle adolescence (around 16–18 years, final secondary school grades). Additionally, it would be helpful to accurately identify at what age university students most frequently adopt OHA from the following sources: *DMD*, *self-learning*, *friends*, and *media & internet*. Second, it would be advisable to conduct longitudinal studies of OHA sources among smaller groups of respondents, tracking the same cohort from earlier stages (such as middle childhood) to the later stages (through late adolescence). This research could enable the identification of periods when specific OH sources exert the most decisive influence, as well as potential “sensitive” phases after which their impact diminishes. Additionally, this approach would be helpful in understanding the interactive effects and influences of different OH sources. Third, it is recommended that researchers investigate potential secondary and other remaining OH sources, using a larger number of items to develop high-quality summary scales for all OH activity sources. Closely related to this recommendation is the development of all the OHAQ-S measures, with particular attention to the conducting of the *construct validation* procedures. Fourth, it is advised to analyse the relationships between OH sources (OHAQ-S measure) and individual measures from the OHAQ questionnaire (*BOHA–basic OH activities*, *ROTB–regularity of tooth brushing*, *ODMD–orientation to DMD,* and *FLOSS–dental floss usage*) to accurately identify the possible determinants of each specific aspect of OHA. Additionally, it is recommended that future work determine the correlations between OH sources (OHAQ-S measure) and individual self-reported oral health status measures among university students.

## 5. Conclusions

The primary sources of information influencing OHA behaviour among university students were *kindergarten*, *self-learning*, *parents*, *DMD*, *primary school*, *media*, and *internet* sources. Female students have differently expressed OHA sources compared to male students. However, both female and male students share several common determinants of OHA, like *DMD* and *university (acquired knowledge)* sources, followed by the moderate determinants of *parents source* and two floss-related sources, *self-learning 2 (floss importance*) and *friends (floss-related)*. Female students also rely on their OHA based on *high school education (I learned the most)* and on information obtained in *kindergarten (I learned to brush my teeth)*. The *primary school OHA source* does not significantly influence the overall OHA behaviour of either the female or the male students. Furthermore, compared to other sources, *university sources* were not prominently expressed among students; the authors of this study recommend that is possible (and necessary) to influence the “final” shaping of students’ OHA behaviour at the university level. In the relevant literature, it is already well known that young people rely most on and trust their *parents* and immediate *authorities* (such as *dentists* or *educators*) when shaping their oral health behaviours. Effective programmes promoting the adoption of healthy habits and enhancing young people’s long-term oral health should include the integration of efforts and activities from the fields of health and education, as well as family members.

## Figures and Tables

**Table 1:** Oral Health Activities Sources Questionnaire (OHAQ-S) measures.

*Parents Source* *(PAR)* Scale							
Items	EIGEN	% VAR	Alpha	FS	Mean ± SD		
	1.88	46.9	0.61				
My parents demonstrated how to brush my teeth.				0.81	3.42	±	1.38
My parents brushed my teeth every night before bed until I went to school.				0.54	2.21	±	1.29
My parents showed me how to brush my teeth properly.				0.79	3.40	±	1.35
I first heard about the importance of flossing from my parents.				0.56	2.52	±	1.32
*Dental Medicine Doctor (DMD) source* scale							
Items	EIGEN	% VAR	Alpha	FS	Mean ± SD		
	2.49	49.7	0.74				
My dentist demonstrated to me how to brush my teeth properly.				0.80	2.60	±	1.48
The dentist explained to me the rules for maintaining oral hygiene.				0.80	2.83	±	1.36
A school doctor taught me how to maintain oral hygiene.				0.54	2.14	±	1.15
A dentist or dental assistant educated me about the importance of maintaining oral hygiene.				0.78	2.70	±	1.34
I first heard about the importance of flossing from my dentist.				0.56	2.44	±	1.37
*Primary School (PRS) source* scale							
Items	EIGEN	% VAR	Alpha	FS	Mean ± SD		
	1.96	48.9	0.65				
My PRS teacher taught me how and how often to brush my teeth.				−0.73	2.17	±	1.31
I learned how to maintain dental and oral hygiene in primary (elementary) school.				−0.64	3.04	±	1.28
I learned how to properly maintain dental and oral hygiene in primary school.				−0.68	2.82	±	1.29
My PRS teacher taught me everything I need to know about maintaining oral hygiene.				−0.74	2.15	±	1.16
*Kindergarten (KG) source* variables							
Item					Mean ± SD		
KG1–I remember the story about the Tooth Fairy.					3.96	±	1.29
KG2–I learned how to brush my teeth back in kindergarten.					3.66	±	1.39
*High school (HS) source* variables							
HS1–I learned the most about maintaining oral hygiene in high school.					2.10	±	1.12
HS2–I learned how to maintain dental and oral hygiene in high school.					1.83	±	1.02
*Self-learning (SL) source* variables							
SL1–I learned most about maintaining oral hygiene completely on my own.					3.34	±	1.16
SL2–I myself realized the importance of flossing.					2.83	±	1.28
*Media & Internet (MI) source* variables							
MI1–I learned most about maintaining oral hygiene from the media or the internet.					2.49	±	1.16
MI2–I learned the most about maintaining oral hygiene from health magazines					2.16	±	1.07
*Friends (FR) source* variables							
FR1–My friends gave me the best information about oral hygiene.					2.01	±	1.16
FR2–I first heard about the importance of flossing from my friends.					1.97	±	1.19
*University (UN) source* variables							
UN1–I gained the knowledge necessary to maintain oral hygiene at university.					1.69	±	1.10
UN2–I was fully educated about the importance of maintaining oral hygiene at university.					1.93	±	1.25

Notes: EIGEN—eigen value, component variance; % VAR—percentage of variance of component; Alpha—Cronbach’s alpha coefficient; FS—factor saturation by component; SD—standard deviation.

**Table 2:** Sensitivity and gender differences of OHA sources.

Variables	Female Students (N = 439)						Male Students (N = 219)						*t*-Test	*p=*
	Mean	±	SD	Skew	Kurt	K–S D	Mean	±	SD	Skew	Kurt	K–S D		
Parents scale	2.91	±	0.94	0.04	−0.46	0.06	2.85	±	0.85	−0.09	−0.45	0.10 *	0.79	0.43
KG1–Tooth fairy	4.06	±	1.30	−1.19	0.18	0.33 *	3.77	±	1.26	−0.63	−0.74	0.25 *	2.70	0.007
KG2–Learned to brush	3.73	±	1.39	−0.73	−0.75	0.25 *	3.53	±	1.39	−0.49	−0.99	0.21 *	1.76	0.08
DMD scale	2.53	±	0.95	0.32	−0.51	0.08 *	2.57	±	0.93	0.18	−0.70	0.06	−0.47	0.64
Primary school scale	2.58	±	0.91	0.41	−0.06	0.08 *	2.48	±	0.83	0.19	0.05	0.09 *	1.38	0.17
HS1–Learned the most	2.12	±	1.13	0.85	−0.01	0.22 *	2.07	±	1.10	0.78	−0.17	0.24 *	0.59	0.56
HS2–Then I learned	1.70	±	0.94	1.22	0.79	0.34 *	2.09	±	1.13	0.77	−0.27	0.24 *	−4.73	<0.001
SL1–Learned the most	3.38	±	1.14	−0.23	−0.64	0.18 *	3.27	±	1.19	−0.22	−0.76	0.16 *	1.16	0.25
SL2–Floss importance	2.96	±	1.29	0.06	−0.98	0.16 *	2.58	±	1.21	0.25	−0.87	0.17 *	3.59	<0.001
MI1–Media & Internet	2.49	±	1.19	0.40	−0.58	0.17 *	2.51	±	1.11	0.20	−0.67	0.21 *	−0.20	0.84
MI2–Health magazines	2.16	±	1.05	0.50	−0.53	0.21 *	2.16	±	1.12	0.62	−0.43	0.23 *	0.02	0.98
FR1–Best information	1.92	±	1.14	1.10	0.39	0.29 *	2.18	±	1.19	0.69	−0.44	0.23 *	−2.70	0.007
FR2–Floss importance	1.92	±	1.15	1.08	0.23	0.30 *	2.09	±	1.26	0.85	−0.38	0.28 *	−1.79	0.07
UN1–Acquired knowledge	1.57	±	1.00	1.82	2.67	0.40 *	1.93	±	1.24	1.14	0.18	0.33 *	−3.93	<0.001
UN2–Fully educated	1.85	±	1.22	1.29	0.53	0.34 *	2.09	±	1.29	0.97	−0.20	0.27 *	−2.35	0.019

Notes: DMD—dental medicine doctor; Mean ± SD—arithmetic mean and standard deviation; Skew—coefficient of asymmetry of the distribution; Kurt—coefficient of kurtosis of the distribution; K–S D—Kolmogorov–Smirnov *goodness-of-fit* test; * significant K–S D test coefficient; *t*-test—*t*-test coefficient; *p=*—significance of the *t*-test coefficient.

**Table 3:** Hierarchical multiple regression analysis of the OHAQ overall score among female students.

Variables	Model 1– Early Sources			Model 2– “Middle” Sources			Model 3– Other Sources			Tolerance
	BETA	t (438)	*p*=	BETA	t (438)	*p*=	BETA	t (438)	*p*=	
Intercept	–	17.34	<0.001	–	12.90	<0.001	–	7.22	<0.001	–
Parents scale	0.23	4.80	<0.001	0.13	2.69	0.007	0.11	2.33	0.020	0.78
KG1–Tooth fairy	0.01	0.14	0.89	0.03	0.65	0.51	0.06	1.38	0.17	0.89
KG2–Learned to brush	0.08	1.85	0.07	0.11	2.37	0.018	0.10	2.27	0.024	0.87
DMD scale	–	–	–	0.37	7.84	<0.001	0.36	7.84	<0.001	0.77
Primary school scale	–	–	–	−0.10	−2.21	0.028	−0.03	−0.67	0.50	0.79
HS1–Learned the most	–	–	–	0.06	1.32	0.19	009	2.12	0.035	0.83
HS2–Then I learned	–	–	–	−0.04	−0.77	0.44	−0.05	−1.07	0.29	0.71
SL1–Learned the most	–	–	–	–	–	–	0.07	1.70	0.09	0.90
SL2–Floss importance	–	–	–	–	–	–	0.27	6.70	<0.001	0.96
MI1–Media & internet	–	–	–	–	–	–	−0.05	−1.18	0.24	0.79
MI2–Health magazines	–	–	–	–	–	–	−0.01	−0.23	0.82	0.75
FR1–Best information	–	–	–	–	–	–	−0.08	−1.74	0.08	0.74
FR2–Floss importance	–	–	–	–	–	–	−0.10	−2.38	0.018	0.83
UN1–Acquired knowledge	–	–	–	–	–	–	0.22	4.05	<0.001	0.56
UN2–Fully educated	–	–	–	–	–	–	−0.03	−0.50	0.62	0.57
R	0.270			0.446			0.571			
R^2^	0.073			0.199			0.326			
Adjusted R^2^	0.067			0.186			0.302			
F (df)	11.44 (3435)			15.25 (7431)			13.61 (15,423)			
*p*=	<0.001			<0.001			<0.001			
R^2^ change	0.073			0.125			0.127			
F change (df)	11.44 (3435)			16.86 (4431)			9.96 (8423)			
F change *p*=	<0.001			<0.001			<0.001			

Notes: BETA—regression coefficient; R—multiple correlation coefficient; R^2^—coefficient of determination; Adjusted R^2^—adjusted multiple determination coefficient; F—coefficient of significance of multiple regression; df—degrees of freedom; *p*=—level of coefficient of significance of multiple regression.

**Table 4:** Hierarchical multiple regression analysis of the OHAQ overall score among male students.

Variables	Model 1– Early Sources			Model 2– “Middle” Sources			Model 3– Other Sources			Tolerance
	BETA	t (218)	*p*=	BETA	t (218)	*p*=	BETA	t (218)	*p*=	
Intercept	–	11.57	<0.001	–	8.44	<0.001	–	5.92	<0.001	–
Parents scale	0.30	4.42	<0.001	0.21	2.90	0.004	0.19	2.70	0.008	0.65
KG1–Tooth fairy	0.00	0.03	0.98	0.02	0.24	0.81	0.03	0.54	0.59	0.90
KG2–Learned to brush	0.08	1.19	0.23	0.06	0.94	0.35	0.07	1.13	0.26	0.76
DMD scale		–	–	0.34	4.96	<0.001	0.29	4.30	<0.001	0.71
Primary school scale		–	–	−0.04	−0.60	0.55	0.01	−0.21	0.63	0.74
HS1–Learned the most		–	–	0.10	1.49	0.14	0.13	1.90	0.06	0.75
HS2–Then I learned		–	–	−0.08	−1.18	0.24	−0.13	−1.82	0.07	0.63
SL1–Learned the most		–	–	–	–	–	0.07	1.23	0.22	0.90
SL2–Floss importance		–	–	–	–	–	0.17	2.58	0.011	0.80
MI1–Media & internet		–	–	–	–	–	−0.03	−0.55	0.58	0.83
MI2–Health magazines		–	–	–	–	–	0.01	0.15	0.88	0.70
FR1–Best information		–	–	–	–	–	−0.01	−0.13	0.90	0.69
FR2–Floss importance		–	–	–	–	–	−0.14	−2.00	0.047	0.65
UN1–Acquired knowledge		–	–	–	–	–	0.30	3.73	<0.001	0.51
UN2–Fully educated		–	–	–	–	–	−0.04	−0.44	0.66	0.43
R	0.340			0.479			0.584			
R^2^	0.116			0.229			0.341			
Adjusted R^2^	0.104			0.204			0.291			
F (df)	9.39 (3215)			8.98 (7211)			7.00 (15,203)			
*p*=	<0.001			<0.001			<0.001			
R^2^ change	0.116			0.114			0.111			
F change (df)	9.39 (3215)			7.77 (4211)			4.28 (8203)			
F change *p*=	<0.001			<0.001			<0.001			

Notes: BETA—regression coefficient; R—multiple correlation coefficient; R^2^—coefficient of determination; Adjusted R^2^—adjusted multiple determination coefficient; F—coefficient of significance of multiple regression; df—degrees of freedom; *p*=—level of coefficient of significance of multiple regression.

**Table 5:** Analysis of variance of the OHA sources by the OHA types among female students.

Variables	OHAQ TYPES MEMBERSHIP								F	*p=*	ƞ^2^
	Excellent (N = 86)		Good (N = 120)		Satisfactory (N = 120)		Poor (N = 113)				
	Mean	SD	Mean	SD	Mean	SD	Mean	SD			
Parents scale	3.12	1.07	2.90	0.97	2.99	0.87	2.67	0.82	4.25	0.006	0.03
KG1–Tooth fairy	4.07	1.40	4.26	1.23	3.93	1.23	3.97	1.35	1.49	0.22	0.01
KG2–Learned to brush	3.95	1.37	3.89	1.40	3.66	1.26	3.45	1.47	2.96	0.032	0.02
DMD scale	3.00	1.04	2.52	0.98	2.55	0.90	2.16	0.72	14.02	<0.001	0.10
Primary school scale	2.59	1.09	2.61	0.92	2.54	0.75	2.58	0.90	0.14	0.94	0.00
HS1–Learned the most	2.35	1.28	2.06	1.03	2.11	1.10	2.04	1.14	1.51	0.21	0.01
HS2–Then I learned	1.76	1.03	1.56	0.82	1.77	0.99	1.73	0.93	1.24	0.29	0.01
SL1–Learned the most	3.49	1.19	3.47	1.25	3.23	1.06	3.36	1.07	1.16	0.33	0.01
SL2–Floss importance	3.59	1.42	2.58	1.16	3.28	1.14	2.52	1.20	19.22	<0.001	0.13
MI1–Media & internet	2.38	1.27	2.41	1.18	2.72	1.14	2.41	1.17	2.07	0.10	0.01
MI2–Health magazines	2.21	1.17	2.02	1.06	2.34	1.00	2.09	0.98	2.20	0.09	0.02
FR1–Best information	1.93	1.26	1.59	0.87	2.07	1.14	2.12	1.21	5.38	0.001	0.04
FR2–Floss importance	1.78	1.12	1.82	1.19	2.02	1.13	2.00	1.15	1.22	0.30	0.01
UN1–Acquired knowledge	1.91	1.30	1.46	1.01	1.64	0.96	1.37	0.64	5.62	0.001	0.04
UN2–Fully educated	2.20	1.48	1.73	1.16	1.86	1.18	1.70	1.05	3.32	0.020	0.02

Notes: SD—standard deviation; F—coefficient of *One-way ANOVA*; *p=*—significance level of the F coefficient; ƞ^2^—eta squared effect size coefficient.

**Table 6:** Analysis of variance of the OHA sources by the OHA types among male students.

Variables		OHAQ Type Membership							F	*p=*	ƞ^2^
	Excellent (N = 20)		Good (N = 71)		Satisfactory (N = 66)		Poor (N = 62)				
	Mean	SD	Mean	SD	Mean	SD	Mean	SD			
Parents scale	3.41	0.82	2.94	0.81	2.94	0.79	2.47	0.84	8.16	<0.001	0.11
KG1–Tooth fairy	4.20	1.20	3.85	1.25	3.59	1.19	3.74	1.37	1.31	0.27	0.02
KG2–Learned to brush	4.05	1.47	3.75	1.36	3.45	1.22	3.18	1.51	2.97	0.033	0.04
DMD scale	3.33	1.02	2.52	0.87	2.83	0.80	2.08	0.85	14.02	<0.001	0.19
Primary school scale	2.51	1.18	2.39	0.79	2.69	0.63	2.34	0.89	2.37	0.07	0.03
HS1–Learned the most	2.00	1.17	2.04	1.02	2.47	1.13	1.69	1.02	5.71	0.001	0.08
HS2–Then I learned	2.00	1.17	1.99	1.04	2.52	1.15	1.79	1.10	5.05	0.002	0.07
SL1–Learned the most	3.30	1.26	3.30	1.16	3.14	1.11	3.37	1.31	0.43	0.73	0.01
SL2–Floss importance	3.15	1.39	2.55	1.18	2.97	1.01	2.02	1.18	9.13	<0.001	0.13
MI1–Media & internet	2.30	0.98	2.52	1.17	2.55	1.00	2.52	1.21	0.26	0.85	0.00
MI2–Health magazines	2.30	1.34	2.08	1.04	2.62	1.16	1.71	0.91	7.92	<0.001	0.11
FR1–Best information	2.15	1.23	2.11	1.18	2.47	1.11	1.97	1.23	2.09	0.10	0.03
FR2–Floss importance	1.55	1.00	2.07	1.27	2.50	1.34	1.85	1.11	4.51	0.004	0.06
UN1–Acquired knowledge	2.35	1.63	1.61	1.06	2.48	1.29	1.56	0.99	9.55	<0.001	0.13
UN2–Fully educated	2.20	1.58	1.89	1.12	2.58	1.36	1.77	1.18	5.26	0.002	0.07

Notes: SD—standard deviation; F—coefficient of *One-way ANOVA*; *p=*—significance level of the F coefficient; ƞ^2^—eta squared effect size coefficient.

**Table 7:** Discriminative analysis of the OHA sources by the OHA types among female students.

DF	λ	Rc	Wilks’ λ	*χ^2^*	df	*p=*
1	0.31	0.49	0.68	168.23 ***	48	<0.001
2	0.10	0.30	0.89	51.57 **	28	0.004
3	0.03	0.17	0.97	12.32	14	0.50
Variables			Structure matrix			
			DF 1	DF 2	DF 3	
Parents scale			0.30	−0.01	−0.26	
KG1–Tooth fairy			0.01	0.32	−0.13	
KG2–Learned to brush			0.19	0.28	−0.18	
DMD scale			0.55	0.14	0.00	
Primary school scale			−0.01	0.10	0.05	
HS1–Learned the most			0.17	−0.01	0.23	
HS2–Then I learned			0.05	−0.27	0.19	
SL1–Learned the most			0.04	0.25	0.22	
SL2–Floss importance			0.59	−0.51	0.03	
MI1–Media & internet			0.02	−0.29	−0.46	
MI2–Health magazines			0.11	−0.34	−0.17	
FR1–Best information			−0.05	−0.56	0.46	
FR2–Floss importance			−0.09	−0.24	−0.09	
UN1–Acquired knowledge			0.34	−0.12	0.14	
UN2–Fully educated			0.26	−0.06	0.28	
OHAQ Types			Group centroids			
			DF 1	DF 2	DF 3	
Excellent			0.93	0.06	0.19	
Good			−0.14	0.44	−0.13	
Satisfactory			0.14	−0.38	−0.18	
Poor			−0.71	−0.11	0.18	

Notes: DF—discriminant function; λ—eigenvalue of the discriminant function; Rc—canonical correlation coefficient; Wilks’ **λ**—Wilks lambda coefficient of the discrimination function; χ^2^— discriminant function significance hi-square test; ***—DF significance level of *p* < 0.001; **—DF significance level of *p* < 0.01; df—degrees of freedom; *p*=—level of statistical significance of DF.

**Table 8:** Discriminative analysis of the OHA sources by the OHA types among male students.

DF	λ	Rc	Wilks’ Lambda	*χ^2^*	SS	*p*=
1	0.44	0.55	0.57	117.90 ***	45	<0.001
2	0.17	0.38	0.82	42.11 *	28	0.042
3	0.05	0.21	0.96	9.45	13	0.74
VARIABLES			Structure matrix			
			DF 1	DF 2	DF 3	
Parents scale			0.46	−0.30	−0.35	
KG1–Tooth fairy			0.03	−0.32	0.02	
KG2–Learned to brush			0.20	−0.31	−0.42	
DMD scale			0.66	−0.16	0.02	
Primary school scale			0.22	0.25	0.09	
HS1–Learned the most			0.34	0.38	−0.33	
HS2–Then I learned			0.30	0.42	−0.09	
SL1–Learned the most			−0.09	−0.12	0.05	
SL2–Floss importance			0.53	0.06	−0.17	
MI1–Media & internet			−0.04	0.12	−0.12	
MI2–Health magazines			0.45	0.35	−0.19	
FR1–Best information			0.20	0.26	−0.06	
FR2–Floss importance			0.14	0.53	−0.38	
UN1–Acquired knowledge			0.48	0.35	0.51	
UN2–Fully educated			0.34	0.37	0.17	
OHAQ Types			Group centroids			
			DF 1	DF 2	DF 3	
Excellent			1.12	−0.92	0.29	
Good			−0.12	−0.21	−0.29	
Satisfactory			0.59	0.50	0.04	
Poor			−0.85	0.01	0.20	

Notes: DF—discriminant function; λ—eigenvalue of the discriminant function; Rc—canonical correlation coefficient; Wilks’ **λ**—Wilks lambda coefficient of the discrimination function; χ^2^—discriminant function significance hi-square test; ***—DF significance level of *p* < 0.001; *—DF significance level of *p* < 0.05; df—degrees of freedom; *p*=—level of statistical significance of DF.

## Data Availability

The raw data supporting the conclusions of this article can be made available by the authors, without undue reservation, upon written request.

## Abbreviations

The following abbreviations are used in this manuscript:

OH	Oral health
OHA	Oral health activities
OH types	Oral health types
OHAQ	Oral health activities questionnaire
OHAQ-S	Oral health activities sources questionnaire
BOHA	Basic oral hygiene activities scale
ROTB	Regularity of tooth brushing scale
FLOSS	The use of dental floss
DMD (DMDO)	Dental medical doctor; also, the corresponding scale in this study
WHO	World Health Organisation
HBSC	Health behaviour in school-aged children
PCA	Principal component analysis
HMRA	Hierarchical multiple regression analysis
DF	Discriminant function
RCT	Randomised control trials
